# 6-Meth­oxyisobenzofuran-1(3*H*)-one

**DOI:** 10.1107/S1600536812039074

**Published:** 2012-09-26

**Authors:** Jorge L. Pereira, Róbson R. Teixeira, Silvana Guilardi, Drielly A. Paixão

**Affiliations:** a Departamento de Química–UFV, Viçosa, MG, Brazil; b Instituto de Química–UFU, Uberlândia, MG, Brazil

## Abstract

In the title compound, C_9_H_8_O_3_, the mol­ecular skeleton is almost planar [r.m.s. deviation = 0.016 (2) Å]. Weak inter­molecular C—H⋯O and C—H⋯π inter­actions consolidate the crystal packing, with the mol­ecules stacking in the [101] direction.

## Related literature

For the biological activity of isobenzofuran-1(*3H*)-one, see: Brady *et al.* (2000 ▶); Huang *et al.* (2012) ▶; Cardozo *et al.* (2005 ▶); Yoganathan *et al.* (2003 ▶); Demuner *et al.* (2006 ▶). For related structures, see: Sun *et al.* (2009 ▶); Mendenhall *et al.* (2003 ▶).
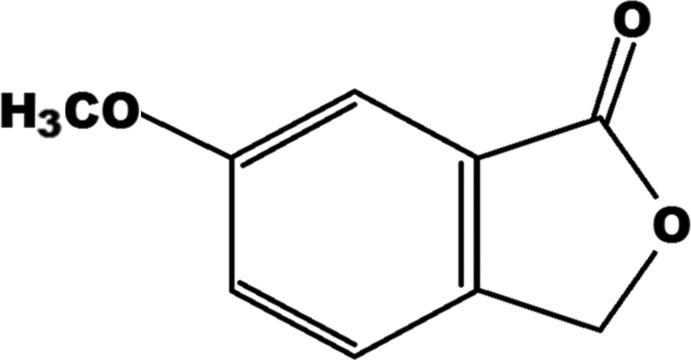


## Experimental

### 

#### Crystal data

C_9_H_8_O_3_
*M* *_r_* = 164.15
Monoclinic, 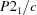

*a* = 9.2922 (19) Å
*b* = 8.4982 (12) Å
*c* = 9.786 (2) Å
β = 90.471 (15)°
*V* = 772.8 (2) Å^3^
*Z* = 4
Mo *K*α radiation
μ = 0.11 mm^−1^
*T* = 293 K
0.28 × 0.17 × 0.12 mm

#### Data collection

Nonius KappaCCD diffractometer
3304 measured reflections
1741 independent reflections
1285 reflections with *I* > 2σ(*I*)
*R*_int_ = 0.036

#### Refinement

*R*[*F* ^2^ > 2σ(*F* ^2^)] = 0.050
*wR*(*F* ^2^) = 0.166
*S* = 1.12
1741 reflections
109 parameters
H-atom parameters constrained
Δρ_max_ = 0.20 e Å^−3^
Δρ_min_ = −0.18 e Å^−3^

### 

Data collection: *COLLECT* (Nonius, 2000 ▶); cell refinement: *DENZO-SMN* (Otwinowski & Minor, 1997 ▶); data reduction: *DENZO-SMN*; program(s) used to solve structure: *SHELXS97* (Sheldrick, 2008 ▶); program(s) used to refine structure: *SHELXL97* (Sheldrick, 2008 ▶); molecular graphics: *ORTEP-3 for Windows* (Farrugia, 1997 ▶); software used to prepare material for publication: *WinGX* (Farrugia, 1999 ▶).

## Supplementary Material

Crystal structure: contains datablock(s) I, global. DOI: 10.1107/S1600536812039074/cv5338sup1.cif

Structure factors: contains datablock(s) I. DOI: 10.1107/S1600536812039074/cv5338Isup2.hkl

Supplementary material file. DOI: 10.1107/S1600536812039074/cv5338Isup3.cml

Additional supplementary materials:  crystallographic information; 3D view; checkCIF report

## Figures and Tables

**Table 1:** Hydrogen-bond geometry (Å, °) *Cg*1 is the centroid of C2–C7 ring.

*D*—H⋯*A*	*D*—H	H⋯*A*	*D*⋯*A*	*D*—H⋯*A*
C9—H9*B*⋯O2^i^	0.96	2.55	3.490 (2)	165
C8—H8*B*⋯*Cg*1^ii^	0.97	2.84	3.637 (2)	140
C9—H9*C*⋯*Cg*1^iii^	0.96	2.91	3.744 (2)	146

Symmetry codes: (i) 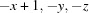; (ii) 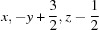; (iii) 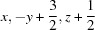.

**Fig. 1.**
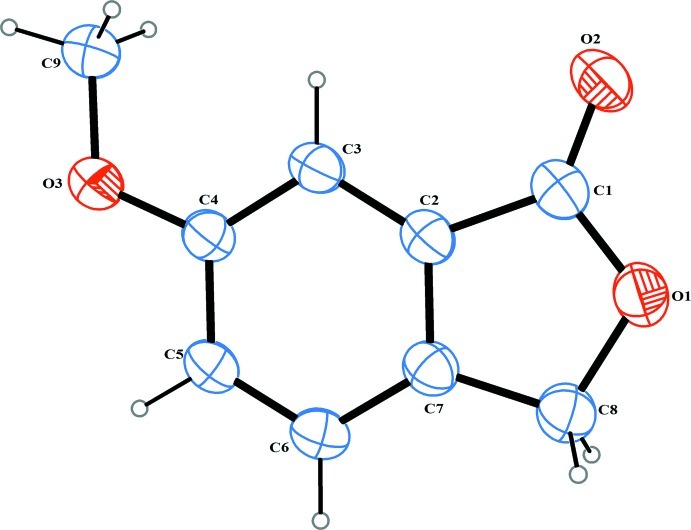
The molecular structure of the title compound, showing the atom labeling and displacement ellipsoids at the 30% probability level.

**Fig. 2.**
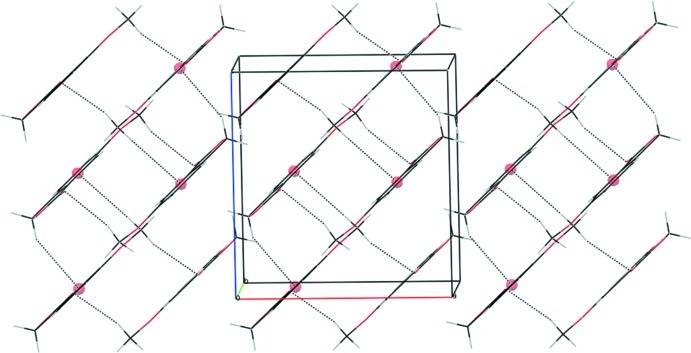
A portion of the crystal packing viewed approximately down the c axis. Dotted lines denote intermolecular C—H···O and C—H···π interactions. Centroids of six-membered rings are drawn as red balls.

## supplementary crystallographic information

### Comment

Isobenzofuran-1(*3H*)-one fragment, γ-lactone fused to aromatic ring, occurs in several compounds that exhibit biological activities ranging from antibacterial (Brady *et al.*, 2000), antioxidant (Zhu *et al.*, 2012), anticonvulsant (Cardozo *et al.*, 2005), and anti-HIV (Yoganathan *et al.*, 2003) to inhibition of the photosynthetic electron transport (Demuner *et al.*, 2006). In a research program devoted to finding the phytotoxic compounds containing the isobenzofuran-1(*3H*)-one core, the title compound was synthesized.

The title molecule (Fig. 1) is essentially planar with a mean deviation of 0.016 (2) Å from the best plane formed by 12 non-H atoms. All bond lengths and angles are normal and correspond to those observed in the related compounds (Sun *et al.*, 2009; Mendenhall *et al.*, 2003). The crystal packing is stabilized by weak intermolecular C—H···O and C—H···π interactions (Table 1, Fig. 2), with a stacking direction of the molecules in [101].

### Experimental

Starting materials were commercially available and were used without further purification. The synthesis of the title compound was carried out as follows. A tube of 40 ml equipped with a magnetic stir bar was charged with Palladium (II) acetate (156.8 mg, 0.70 mmol), potassium dihydrogen phosphate (3654 mg, 21.0 mmol), 3-methoxy benzoic acid (1064 mg, 7.00 mmol) and dibromomethane (28 ml). The tube was sealed with a teflon cap and the reaction mixture was stirred at 140 °C for 36 h. After this time, the mixture was filtered through a pad of celite. The filtrate was concentrated under reduced pressure and the residue was purified by silica gel column chromatography (hexane-ethyl acetate 2:1 *v*/*v*) to afford the title compound in 59% yield (681 mg, 4.15 mmol) as a white solid. The crystals suitable for X-ray crystallographic analysis were obtained by slow evaporation from acetone solution at room temperature. *M*.p. 116.9–118.4 °C. IR (selected bands, cm^-1^): 3003, 2925, 2837, 1733, 1600, 1585, 1488, 1454, 1431, 1267, 1206, 1028, 973, 869, 749, 690, 545. ^1^H NMR (300 MHz, MeOH-*d_4_*) δ 3.87 (s, 3H, H-9), 5.26 (s, 2H, H-8), 7.22–7.38 (m, 3H, H-3, H-5 and H-6); ^13^C NMR (75 MHz, MeOH-*d_4_*) δ 56.0 (C-9), 69.7 (C-8), 107.7 (C-3), 123.1; 123.3 (C-5 and C-6), 127.3 (C-2), 139.1 (C-7), 160.8 (C-4), 171.4 (C-1). HREIMS *m/z* (*M*+H^+^): Calcd for C_9_H_8_O_3_, 165.0552; found: 165.0608.

### Crystal data

**Table:**

C_9_H_8_O_3_	*F*(000) = 344
*M**_r_* = 164.15	*D*_x_ = 1.411 Mg m^−^^3^
Monoclinic, *P*2_1_/*c*	Mo *K*α radiation, λ = 0.71073 Å
*a* = 9.2922 (19) Å	Cell parameters from 3082 reflections
*b* = 8.4982 (12) Å	θ = 2.1–27.5°
*c* = 9.786 (2) Å	µ = 0.11 mm^−^^1^
β = 90.471 (15)°	*T* = 293 K
*V* = 772.8 (2) Å^3^	Prism, colourless
*Z* = 4	0.28 × 0.17 × 0.12 mm

### Data collection

**Table:**

Nonius KappaCCD diffractometer	1285 reflections with *I* > 2σ(*I*)
Radiation source: Enraf Nonius FR590	*R*_int_ = 0.036
Graphite monochromator	θ_max_ = 27.5°, θ_min_ = 2.2°
Detector resolution: 9 pixels mm^-1^	*h* = −12→12
CCD rotation images, thick slices scans	*k* = −10→11
3304 measured reflections	*l* = −12→12
1741 independent reflections	

### Refinement

**Table:**

Refinement on *F*^2^	Primary atom site location: structure-invariant direct methods
Least-squares matrix: full	Secondary atom site location: difference Fourier map
*R*[*F*^2^ > 2σ(*F*^2^)] = 0.050	Hydrogen site location: inferred from neighbouring sites
*wR*(*F*^2^) = 0.166	H-atom parameters constrained
*S* = 1.12	*w* = 1/[σ^2^(*F*_o_^2^) + (0.0943*P*)^2^ + 0.032*P*] where *P* = (*F*_o_^2^ + 2*F*_c_^2^)/3
1741 reflections	(Δ/σ)_max_ < 0.001
109 parameters	Δρ_max_ = 0.20 e Å^−^^3^
0 restraints	Δρ_min_ = −0.18 e Å^−^^3^

### Special details

**Table:**

Geometry. All e.s.d.'s (except the e.s.d. in the dihedral angle between two l.s. planes) are estimated using the full covariance matrix. The cell e.s.d.'s are taken into account individually in the estimation of e.s.d.'s in distances, angles and torsion angles; correlations between e.s.d.'s in cell parameters are only used when they are defined by crystal symmetry. An approximate (isotropic) treatment of cell e.s.d.'s is used for estimating e.s.d.'s involving l.s. planes.
Refinement. Refinement of *F*^2^ against ALL reflections. The weighted *R*-factor *wR* and goodness of fit *S* are based on *F*^2^, conventional *R*-factors *R* are based on *F*, with *F* set to zero for negative *F*^2^. The threshold expression of *F*^2^ > σ(*F*^2^) is used only for calculating *R*-factors(gt) *etc*. and is not relevant to the choice of reflections for refinement. *R*-factors based on *F*^2^ are statistically about twice as large as those based on *F*, and *R*- factors based on ALL data will be even larger.

### Fractional atomic coordinates and isotropic or equivalent isotropic displacement parameters (Å^2^)

**Table:**

	*x*	*y*	*z*	*U*_iso_*/*U*_eq_	
O1	0.93834 (12)	0.04009 (14)	0.16871 (11)	0.0695 (4)	
O2	0.79489 (14)	−0.11746 (14)	0.04458 (12)	0.0752 (4)	
O3	0.54600 (12)	0.40633 (13)	−0.19372 (11)	0.0699 (4)	
C1	0.83141 (17)	0.0134 (2)	0.07489 (15)	0.0612 (4)	
C2	0.77893 (16)	0.16711 (17)	0.02593 (13)	0.0552 (4)	
C3	0.67205 (15)	0.19885 (18)	−0.07089 (14)	0.0569 (4)	
H3	0.6212	0.1185	−0.114	0.068*	
C4	0.64497 (16)	0.35569 (18)	−0.09995 (14)	0.0568 (4)	
C5	0.72174 (17)	0.47477 (18)	−0.03217 (16)	0.0632 (4)	
H5	0.7013	0.5794	−0.0524	0.076*	
C6	0.82660 (17)	0.44069 (19)	0.06357 (16)	0.0639 (4)	
H6	0.8766	0.5208	0.108	0.077*	
C7	0.85594 (16)	0.28364 (18)	0.09213 (14)	0.0572 (4)	
C8	0.96195 (18)	0.2080 (2)	0.18656 (16)	0.0663 (5)	
H8A	1.0596	0.2365	0.1624	0.08*	
H8B	0.9448	0.2392	0.2804	0.08*	
C9	0.45913 (19)	0.2893 (2)	−0.25875 (18)	0.0730 (5)	
H9A	0.3942	0.3389	−0.3222	0.11*	
H9B	0.4051	0.2336	−0.191	0.11*	
H9C	0.5197	0.2169	−0.3067	0.11*	

### Atomic displacement parameters (Å^2^)

**Table:**

	*U*^11^	*U*^22^	*U*^33^	*U*^12^	*U*^13^	*U*^23^
O1	0.0805 (7)	0.0576 (7)	0.0702 (7)	0.0088 (5)	−0.0046 (5)	0.0056 (5)
O2	0.0934 (9)	0.0475 (7)	0.0847 (8)	0.0000 (5)	0.0030 (6)	0.0033 (5)
O3	0.0797 (7)	0.0492 (7)	0.0804 (7)	−0.0024 (5)	−0.0172 (6)	0.0022 (5)
C1	0.0700 (9)	0.0536 (9)	0.0602 (8)	0.0018 (7)	0.0085 (7)	0.0018 (6)
C2	0.0635 (8)	0.0479 (8)	0.0541 (7)	0.0004 (6)	0.0070 (6)	0.0000 (6)
C3	0.0651 (8)	0.0457 (8)	0.0599 (8)	−0.0047 (6)	0.0027 (6)	−0.0034 (6)
C4	0.0635 (8)	0.0480 (8)	0.0588 (8)	−0.0014 (6)	−0.0003 (6)	−0.0001 (6)
C5	0.0757 (9)	0.0428 (8)	0.0708 (9)	0.0005 (6)	−0.0035 (7)	−0.0013 (6)
C6	0.0734 (9)	0.0496 (8)	0.0686 (9)	−0.0049 (7)	−0.0032 (7)	−0.0081 (7)
C7	0.0633 (8)	0.0524 (9)	0.0561 (7)	−0.0001 (6)	0.0046 (6)	−0.0021 (6)
C8	0.0728 (9)	0.0610 (10)	0.0649 (9)	0.0037 (7)	−0.0022 (7)	−0.0042 (7)
C9	0.0790 (10)	0.0593 (10)	0.0805 (10)	−0.0097 (8)	−0.0147 (8)	0.0019 (8)

### Geometric parameters (Å, º)

**Table:**

O1—C1	1.366 (2)	C5—C6	1.377 (2)
O1—C8	1.454 (2)	C5—H5	0.93
O2—C1	1.199 (2)	C6—C7	1.390 (2)
O3—C4	1.3635 (19)	C6—H6	0.93
O3—C9	1.427 (2)	C7—C8	1.491 (2)
C1—C2	1.473 (2)	C8—H8A	0.97
C2—C7	1.380 (2)	C8—H8B	0.97
C2—C3	1.393 (2)	C9—H9A	0.96
C3—C4	1.385 (2)	C9—H9B	0.96
C3—H3	0.93	C9—H9C	0.96
C4—C5	1.402 (2)		
			
C1—O1—C8	110.59 (12)	C5—C6—H6	120.8
C4—O3—C9	117.14 (13)	C7—C6—H6	120.8
O2—C1—O1	121.50 (16)	C2—C7—C6	119.63 (15)
O2—C1—C2	130.52 (17)	C2—C7—C8	108.60 (14)
O1—C1—C2	107.98 (14)	C6—C7—C8	131.77 (14)
C7—C2—C3	122.97 (15)	O1—C8—C7	104.50 (12)
C7—C2—C1	108.34 (15)	O1—C8—H8A	110.9
C3—C2—C1	128.69 (14)	C7—C8—H8A	110.9
C4—C3—C2	116.94 (14)	O1—C8—H8B	110.9
C4—C3—H3	121.5	C7—C8—H8B	110.9
C2—C3—H3	121.5	H8A—C8—H8B	108.9
O3—C4—C3	124.20 (14)	O3—C9—H9A	109.5
O3—C4—C5	115.37 (14)	O3—C9—H9B	109.5
C3—C4—C5	120.43 (15)	H9A—C9—H9B	109.5
C6—C5—C4	121.64 (15)	O3—C9—H9C	109.5
C6—C5—H5	119.2	H9A—C9—H9C	109.5
C4—C5—H5	119.2	H9B—C9—H9C	109.5
C5—C6—C7	118.38 (15)		
			
C8—O1—C1—O2	179.83 (13)	O3—C4—C5—C6	−178.97 (12)
C8—O1—C1—C2	−0.01 (16)	C3—C4—C5—C6	0.7 (2)
O2—C1—C2—C7	−179.93 (15)	C4—C5—C6—C7	0.2 (2)
O1—C1—C2—C7	−0.11 (16)	C3—C2—C7—C6	0.6 (2)
O2—C1—C2—C3	−0.6 (3)	C1—C2—C7—C6	179.99 (12)
O1—C1—C2—C3	179.23 (12)	C3—C2—C7—C8	−179.21 (12)
C7—C2—C3—C4	0.2 (2)	C1—C2—C7—C8	0.18 (16)
C1—C2—C3—C4	−179.01 (12)	C5—C6—C7—C2	−0.8 (2)
C9—O3—C4—C3	4.2 (2)	C5—C6—C7—C8	178.95 (14)
C9—O3—C4—C5	−176.13 (13)	C1—O1—C8—C7	0.11 (15)
C2—C3—C4—O3	178.74 (12)	C2—C7—C8—O1	−0.18 (15)
C2—C3—C4—C5	−0.9 (2)	C6—C7—C8—O1	−179.96 (14)

### Hydrogen-bond geometry (Å, º)

Cg1 is the centroid of C2–C7 ring.

**Table:**

*D*—H···*A*	*D*—H	H···*A*	*D*···*A*	*D*—H···*A*
C9—H9*B*···O2^i^	0.96	2.55	3.490 (2)	165
C8—H8*B*···*Cg*1^ii^	0.97	2.84	3.637 (2)	140
C9—H9*C*···*Cg*1^iii^	0.96	2.91	3.744 (2)	146

Symmetry codes: (i) −*x*+1, −*y*, −*z*; (ii) *x*, −*y*+3/2, *z*−1/2; (iii) *x*, −*y*+3/2, *z*+1/2.
